# Stretchable, Adhesive, and Biocompatible Hydrogel Based on Iron–Dopamine Complexes

**DOI:** 10.3390/polym15224378

**Published:** 2023-11-10

**Authors:** Celine Lee, He-Shin Huang, Yun-Ying Wang, You-Sheng Zhang, Rajan Deepan Chakravarthy, Mei-Yu Yeh, Hsin-Chieh Lin, Jeng Wei

**Affiliations:** 1 Department of Chemistry, Chung Yuan Christian University, No. 200, Zhongbei Rd., Zhongli Dist., Taoyuan City 320314, Taiwan; clys0813@gmail.com (C.L.); candyhuang16@gmail.com (H.-S.H.); g11063013@cycu.org.tw (Y.-Y.W.); brian20010418@gmail.com (Y.-S.Z.); 2 Department of Materials Science and Engineering, National Yang Ming Chiao Tung University, No. 1001, Daxue Rd., East Dist., Hsinchu City 300093, Taiwan; rdciitmadras@gmail.com; 3 Heart Center, Cheng Hsin General Hospital, No. 45, Cheng Hsin St., Beitou Dist., Taipei City 112401, Taiwan

**Keywords:** stretchable, adhesive, hydrogel, polyacrylamide, dopamine

## Abstract

Hydrogels’ exceptional mechanical strength and skin-adhesion characteristics offer significant advantages for various applications, particularly in the fields of tissue adhesion and wearable sensors. Herein, we incorporated a combination of metal-coordination and hydrogen-bonding forces in the design of stretchable and adhesive hydrogels. We synthesized four hydrogels, namely PAID-0, PAID-1, PAID-2, and PAID-3, consisting of acrylamide (AAM), *N,N*′-methylene-bis-acrylamide (MBA), and methacrylic-modified dopamine (DA). The impact of different ratios of iron (III) ions to DA on each hydrogel’s performance was investigated. Our results demonstrate that the incorporation of iron–dopamine complexes significantly enhances the mechanical strength of the hydrogel. Interestingly, as the DA content increased, we observed a continuous and substantial improvement in both the stretchability and skin adhesiveness of the hydrogel. Among the hydrogels tested, PAID-3, which exhibited optimal mechanical properties, was selected for adhesion testing on various materials. Impressively, PAID-3 demonstrated excellent adhesion to diverse materials and, combined with the low cytotoxicity of PAID hydrogel, holds great promise as an innovative option for biomedical engineering applications.

## 1. Introduction

Hydrogels, recognized as soft materials in the field of materials science, are three-dimensional (3D) cross-linked polymer networks with the remarkable capability of absorbing and retaining a substantial amount of water [1,2,3,4,5,6,7]. The significant attention hydrogels have received is due to their high water content and the related potential they hold for numerous biomedical applications, including tissue engineering [8,9,10,11,12], soft robotics [13,14,15,16,17], wearable devices [18,19,20,21], personal care [22,23,24], and more. Hydrogels are commonly prepared using both synthetic and natural polymers. Synthetic hydrogels employ diverse chemistries to cross-link synthetic polymer molecules, resulting in hydrogels that exhibit relatively high mechanical strength. However, once these hydrogels are formed, they become challenging to remold. Consequently, they are not suitable for many applications, particularly in the fields of cell culture and tissue engineering. To address this limitation, the incorporation of physical non-covalent bond forces, such as metal coordination, host-guest interactions, ionic interactions, hydrogen bonding, hydrophobic interactions, and π-π interactions, is employed to enhance the flexibility, adjustability, and reversibility of hydrogels [25,26,27,28,29,30,31].

Inspired by nature, specific biomaterials that possess abundant metal-coordination bonds, for example, mussels, exhibit an impressive blend of high toughness, adhesion, and reversibility [32,33,34,35,36,37]. As a result, this concept of biomaterials is extensively used by researchers in the development of functional hydrogels [38,39,40,41,42,43,44]. Das et al. have developed hydrogels of poly(methacrylamide-*co*-vinylimidazole)-M^2+^ (M = Ni and Zn), which demonstrate an exceptionally broad range of mechanical properties, rapid self-recovery, remarkable fatigue resistance, efficient self-healing, and temperature-dependent shape memory behavior [38]. Hao et al. have devised metal-ligand-coordinated hydrogels with adjustable strength and thermosensitivity [39]. Wu and Zheng have reported a physical hydrogel comprised of poly(acrylamide-*co*-acrylic acid) (P(AAm-*co*-AAc)) cross-linked by carboxyl–Fe^3+^ coordination complexes. This hydrogel exhibits remarkable stiffness, toughness, fatigue resistance, and the ability to undergo stimulation-triggered healing [42]. More recently, Wu et al. have created robust hydrazide-transition metal-coordination complexes to enhance the toughness of hydrogels [43].

Therefore, in this study, we chose to employ the approach of metal coordination to improve the mechanical properties of hydrogels. We specifically opted to utilize dopamine as a building block due to its reputation for forming iron–dopamine complexes [33,44]. Simultaneously, dopamine has the potential to engage in aromatic π-stack interactions with drugs like bortezomib, doxorubicin, topotecan, and others, facilitating controlled drug delivery for cancer treatment [45,46,47]. Furthermore, dopamine holds promise for emulating mussel adhesives, offering excellent adhesion properties. We synthesized four hydrogels, namely PAID-0, PAID-1, PAID-2, and PAID-3, composed of acrylamide (AAM), *N,N*′-methylene-bis-acrylamide (MBA), and methacrylic-modified dopamine (DA). Our investigation focused on evaluating the impact of two factors: (1) the presence of iron–dopamine complexes (PAID-0 served as the control); and (2) the influence of hydrogen-bonding interactions involving dopamine on the mechanical and adhesive properties of the hydrogels. We demonstrated that the incorporation of iron–dopamine complexes substantially enhances the mechanical strength of the hydrogel. Notably, with increasing dopamine content, we observed a continuous and pronounced improvement in both the stretchability and skin-adhesion properties of the hydrogel. This discovery opens up new possibilities for the application of tissue adhesion and wearable sensors.

## 2. Materials and Methods

### 2.1. Materials

Acrylamide and 3-hydroxytyramine (dopamine) hydrochloride were obtained from Acros Organics (Geel, Antwerp, Belgium); iron (III) chloride hexahydrate and *N,N*′-methylenebisacrylamide were sourced from Alfa Aesar (Ward Hill, MA, USA). The compound 2-hydroxy-4′-(2-hydroxyethoxy)-2-methylpropiophenone (Irgacure 2959) was acquired from Sigma-Aldrich (St. Louis, MO, USA). Methacrylic-modified dopamine was synthesized following the protocol outlined in the literature (for further details, refer to the Supporting Information) [48]. Deionized water was utilized in all experiments.

### 2.2. Preparation of Hydrogels

PAID hydrogels were synthesized through the radical polymerization of acrylamide, iron (III) chloride hexahydrate, and methacrylic-modified dopamine in the presence of *N,N*′-methylenebisacrylamide as a cross-linker and Irgacure 2959 (2 mol % to monomer) as a radical initiator in deionized water. The specific compositions of the hydrogels were listed in Table S1 (see Supplementary Materials).

### 2.3. Characterizations

Nuclear magnetic resonance (NMR) spectra were obtained using a Bruker (Billerica, MA, USA) AVANCEII-400 MHz spectrometer, with D_2_O serving as the solvent. The molecular characteristics and interactions of PAID hydrogels were assessed using a Thermo Fisher Scientific (Waltham, MA, USA) Nicolet iS5 Fourier transform infrared (FT-IR) spectrometer, while the morphologies of the hydrogels were examined through scanning electron microscopy (SEM) using the JEOL (Tokyo, Japan) JSM-7600F model. To prepare the test samples, the hydrogels were subjected to freeze-vacuum drying. Briefly, the hydrogel samples were rapidly frozen in liquid nitrogen and subsequently lyophilized using a freeze-dryer system under vacuum conditions at a temperature of −80 °C for a minimum of 24 h, allowing all the water to sublimate.

### 2.4. Mechanical Measurements

The PAID hydrogels underwent tensile testing using a Gotech (Taichung, Taiwan) AI-3000-U tensile tester. Rheological measurements were conducted using a TA rheometer (DHR-1, New Castle, Delaware, USA) equipped with a parallel plate setup. The setup consisted of a 20-millimeter-diameter plate with a 1 mm gap between them. Dynamic oscillatory frequency sweep experiments were performed to determine the shear storage modulus (G′) and loss modulus (G′′) as a function of angular frequency (ω). The measurements were taken across the range of ω from 0.1 to 100 rad/sec at a strain (γ) of 1% and at a temperature of 25 °C.

### 2.5. Biocompatibility of Hydrogels

The biocompatibility of PAID hydrogels was assessed by cultivating L929 cells with an extraction medium. To prepare the extraction medium, PAID-0, PAID-1, and PAID-3 hydrogels were immersed in Dulbecco’s Modified Eagle Medium (DMEM, Gibco, Life Technologies, Carlsbad, CA, USA) at a volume ratio of 1:10 (hydrogel volume to medium volume) and incubated at 37 °C for two days. L929 cells were seeded in 96-well plates and cultured with DMEM at 37 °C under 5% CO_2_ for one day. Subsequently, the DMEM was replaced with the extraction medium, allowing the cells to grow for one, three, and seven days. Cell viability assays were performed on the L929 cells cultured with the extraction medium and DMEM using the MTT reagent. The resulting solution’s optical density was measured at 595 nm using a BioTek (Winooski, VT, USA) 800 TS microplate reader. Cells that were not exposed to the test hydrogels were designated as the control group.

## 3. Results and Discussion

### 3.1. Preparation of PAID Hydrogels

**Scheme 1:**
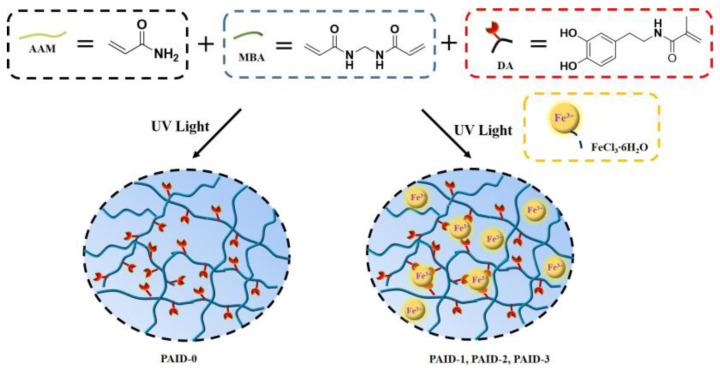
Schematic diagram of PAID hydrogel, where acrylamide is AAM, *N,N*′-methylene-bis-acrylamide is MBA, and methacrylic-modified dopamine is DA.

**Figure 1:**
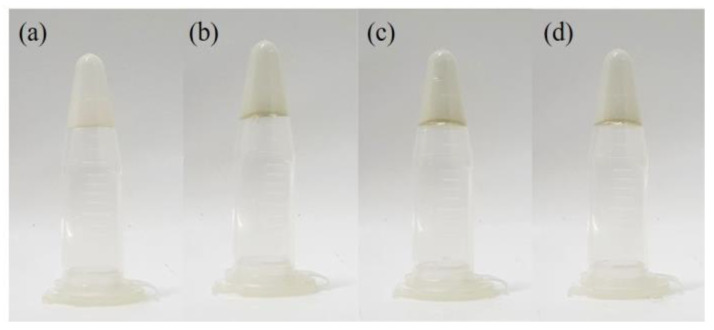
Photographs of (**a**) PAID-0, (**b**) PAID-1, (**c**) PAID-2, and (**d**) PAID-3 hydrogels.

Scheme 1 presents the schematic diagram of the PAID hydrogels. We utilized AAM, MBA, and different ratios of iron (III) ions to DA to prepare the hydrogels, namely PAID-1 (iron ion: DA = 1:3), PAID-2 (iron ion: DA = 1:6), and PAID-3 (iron ion: DA = 1:9); however, continuously increasing the ratio of DA will deteriorate the formability of the hydrogel, resulting in a sticky or pasty consistency. Meanwhile, we also prepared PAID-0 (iron ion: DA = 0:3) for comparison with PAID-1 in order to understand the influence of iron ions on the hydrogel. As shown in Figure 1, the appearance of PAID-0 is a transparent white hydrogel, and, upon the addition of FeCl_3_·6H_2_O, the hydrogels transform into transparent light yellow hydrogels (PAID-1, PAID-2, and PAID-3). These indications suggest that the addition of iron ions may lead to the formation of an iron–DA complex, resulting in a significant change in the appearance of the hydrogel.

### 3.2. Spectral Analysis and Morphologies of PAID Hydrogels

**Figure 2:**
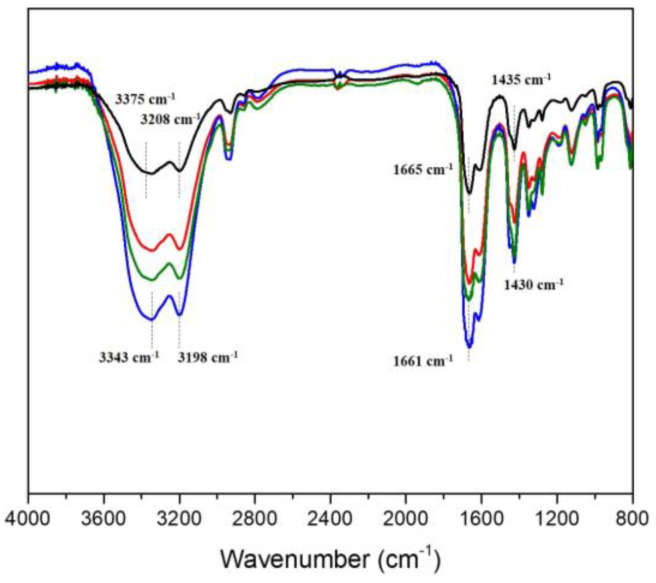
FT-IR spectra of PAID hydrogels. (Black for PAID-0, red for PAID-1, green for PAID-2, and blue for PAID-3).

**Figure 3:**
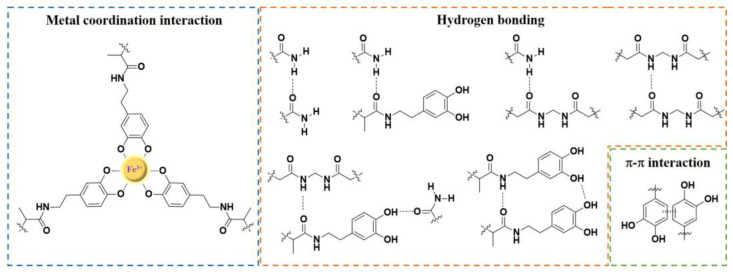
Proposed potential molecular interactions in PAID hydrogels.

**Figure 4:**
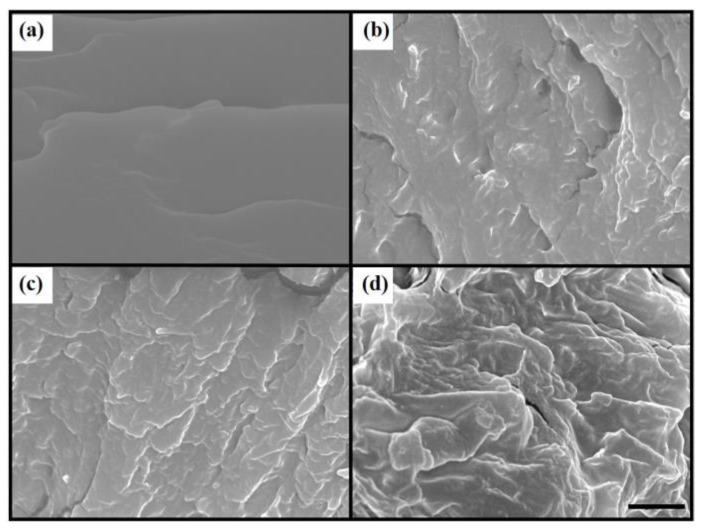
SEM images of (**a**) PAID-0, (**b**) PAID-1, (**c**) PAID-2, and (**d**) PAID-3 hydrogels. (Scale bar: 1 μm).

We employ Fourier transform infrared spectroscopy (FT-IR) to analyze PAID hydrogels and investigate the intermolecular forces between iron ions and polymers (DA components). Through the analysis of the absorbance FT-IR peaks of PAID-0, it was observed that the absorption bands at 3375 and 3208 cm^−1^ were indicative of the N–H and/or O–H stretching vibration [49]. Additionally, the absorption bands around 1665 and 1435 cm^−1^ were characteristic of C=O stretching in the amide group and C=C stretching in aromatics, respectively [50,51]. It is worth noting that the O–H stretching bands manifest at lower frequencies, i.e., 3343 and 3198 cm^−1^, suggesting the potential formation of iron–DA complexes in PAID-1, PAID-2, and PAID-3 (Figure 2) [52]. In the meantime, the C=O and C=C (aromatic) signals likewise shift to lower wavenumbers with an increasing DA content, indicating that the elevated DA content might enhance hydrogen-bonding and π-π-stacking interactions [53]. Moreover, we conducted a comparison between the PAID-0 hydrogel and the AAM/MBA hydrogel described in the literature [54,55]. Our observations revealed that, in contrast to the AAM/MBA hydrogel, the N-H and C=O vibrations in the PAID-0 gel shifted towards lower wavenumbers, possibly due to the enhanced hydrogen bonds resulting from the incorporation of DA. Based on the FT-IR data, we have discerned plausible molecular interactions within PAID hydrogels, involving metal-coordination, hydrogen-bonding, and π-π-stacking interactions (Figure 3). Subsequently, we used a scanning electron microscope (SEM) to examine the nanostructures of the PAID hydrogels. As can be seen in Figure 4, PAID-0 showed a uniform and flat structure, whereas PAID-1, PAID-2, and PAID-3 exhibited distinct layered structures. This observation suggests that the presence of iron ions can possibly trigger the formation of iron–DA complexes. Moreover, as the amount of DA increases, the layered structure becomes more densely packed, implying that the stretchability of the hydrogel can be enhanced as a result. The SEM analysis revealing layered structures in PAID-1, PAID-2, and PAID-3 further strengthens the evidence of the intermolecular forces present in their FT-IR spectra.

### 3.3. Mechanical Properties of PAID Hydrogels

**Figure 5:**
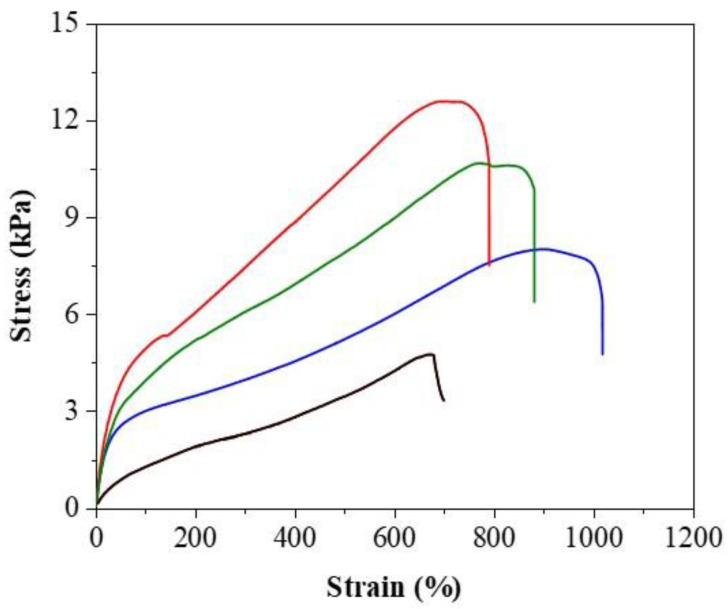
Tensile stress-strain curves of PAID hydrogels. (Black for PAID-0, red for PAID-1, green for PAID-2, and blue for PAID-3).

**Figure 6:**
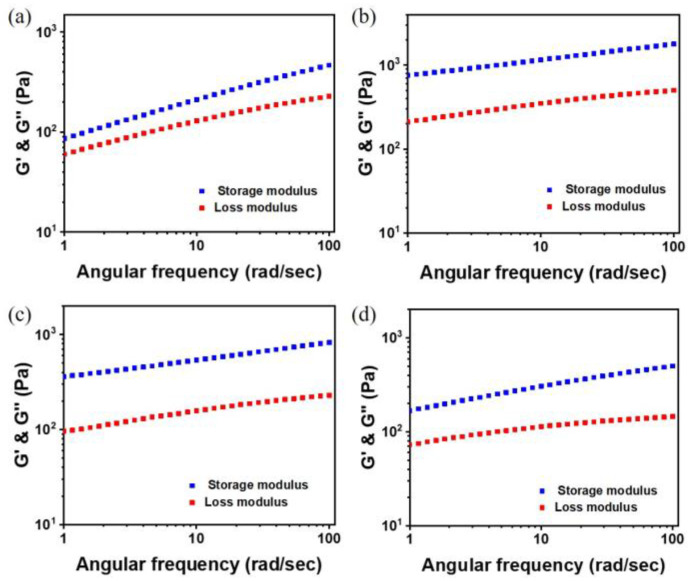
Frequency-dependent (ω = 0.1–100 rad/sec) rheology measurement of (**a**) PAID-0, (**b**) PAID-1, (**c**) PAID-2, and (**d**) PAID-3 hydrogels.

It is known that tensile stress-strain analysis and rheology play crucial roles in various fields of science and engineering. On the one hand, tensile stress-strain analysis helps in designing and assessing the mechanical performance of materials, determining their suitability for specific applications [56,57]. On the other hand, rheology finds widespread applications across diverse fields, including polymer processing, chemical engineering, and pharmaceuticals. The importance of rheology lies in understanding the flow and deformation behavior of materials, which is critical for optimizing processes and developing high-quality products [58,59]. In Figure 5, the tensile stress-strain curves of PAID hydrogels are presented. It was found that PAID-0 exhibited a tensile strength of 4.7 kPa and an elongation break of 675%. Upon the introduction of iron ions into PAID-0, i.e., PAID-1, both the tensile strength and elongation break increased to 12.6 kPa and 743%, respectively. The dramatic enhancement in tensile strength of PAID-1 compared to PAID-0 is likely attributed to the formation of a tris-complex between iron and DA [44]. By keeping the amount of iron ions constant and progressively increasing the amount of DA, we observed a continuous increase in elongation at break; however, there was a decrease in tensile strength. Specifically, for PAID-2 and PAID-3, the tensile strength values were reduced to 10.7 kPa and 8.0 kPa, respectively, while the corresponding elongation break values were measured at 882% and 1011%. The elevated elongation break values in PAID-2 and PAID-3 are probably due to the formation of additional reversible noncovalent bonds, such as hydrogen bonding and/or π-π interactions, ultimately resulting in the enhanced stretchability of the hydrogel. This improvement in mechanical properties aligns with the findings derived from the SEM images presented in Figure 4. The SEM images provided visual evidence that the microstructure of PAID-3 supports higher elongation before failure, which is consistent with the increased elongation break values observed in the mechanical testing. Furthermore, when compared to the metal coordination hydrogels reported in the literature, PAID-3 presented outstanding stretchability, reaching 1011% strain, as indicated in Table S2. This remarkable stretchability opens up possibilities for various applications, particularly in scenarios where a hydrogel with high mechanical flexibility is required, such as in biomedical devices and tissue engineering. These findings highlight the potential of PAID hydrogels to provide innovative solutions in materials science and biotechnology. Figure 6 displays the dynamic rheological behaviors of PAID hydrogels: all of them exhibited a storage modulus (G′) higher than the loss modulus (G″), indicating their viscoelastic solid nature. The values of G′ were determined to be 251 Pa for PAID-0, 1390 Pa for PAID-1, 699 Pa for PAID-2, and 352 Pa for PAID-3. All these hydrogels show G′ values exceeding 100 Pa, which provides sufficient support for cell culture by being able to bear the weight of cells [60].

### 3.4. Biocompatibility of PAID Hydrogels

**Figure 7:**
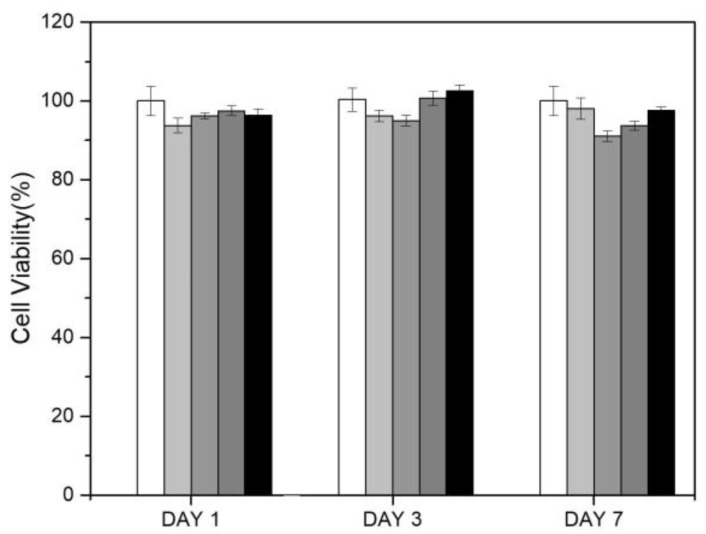
Cell viability of L929 cells on PAID hydrogels for 1, 3, and 7 days. The arrangement from left to right includes the following: control, PAID-0, PAID-1, PAID-2, and PAID-3.

For biomedical applications, it is crucial to assess the biocompatibility of hydrogels [61,62,63,64]. In order to evaluate hydrogels’ compatibility with living cells, the widely-used MTT assay was employed [65,66]. This assay provides valuable insights into the viability and metabolic activity of cells in the presence of the hydrogels, aiding in determining their suitability for use in various biomedical applications such as tissue engineering and drug delivery systems. As depicted in Figure 7, the cell viability of L929 cells on PAID hydrogels was assessed for 1, 3, and 7 days. The cell survival rate of L929 cells cultured in these hydrogels for 7 days was above 90%, confirming the exceptional biocompatibility of PAID hydrogels. The statistical analysis of *p*-values was conducted using the Student’s *t*-test and is summarized in Table S4. The significance of these *p*-values lies in their capacity to determine whether the differences observed between the groups are statistically meaningful. A *p*-value below the conventional threshold of 0.05 indicates that the observed differences are not likely the result of random chance alone, demonstrating a statistically significant outcome. It is well established that polyacrylamide-based gels can undergo degradation under various environmental conditions, including chemical, photolytic, thermal, mechanical, and biological processes. Among them, the biodegradation characteristics of hydrogels play a crucial role in drug delivery and tissue engineering. Therefore, we evaluated the degradation of PAID hydrogels in a phosphate-buffered saline solution at 37 °C; the results are depicted in Figure S1. The degradation rates of the hydrogels follow the order PAID-0 > PAID-3 ≈ PAID-2 > PAID-1, which correlates with their mechanical strength trends. This indicates that metal-coordination interactions may be the predominant factor driving degradation (Figure S1).

### 3.5. Adhesive and Stretchable Capacities of PAID Hydrogels

**Figure 8:**
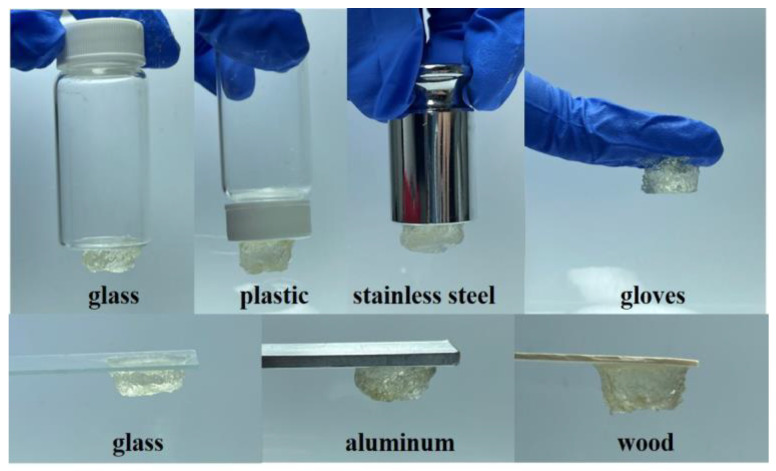
The adhesive properties of the PAID-3 hydrogel were evaluated on various substrates such as glass, plastic, stainless steel, gloves, aluminum, and wood.

**Figure 9:**
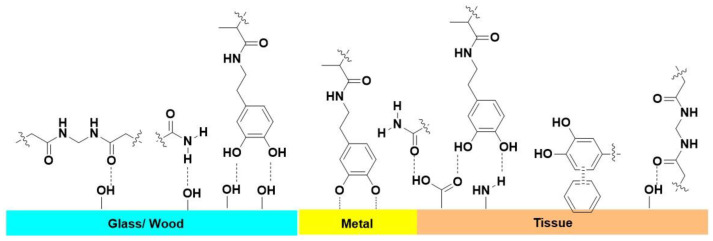
Schematic representation of the interface adhesion mechanism of the PAID-3 hydrogel to various substrates.

**Figure 10:**
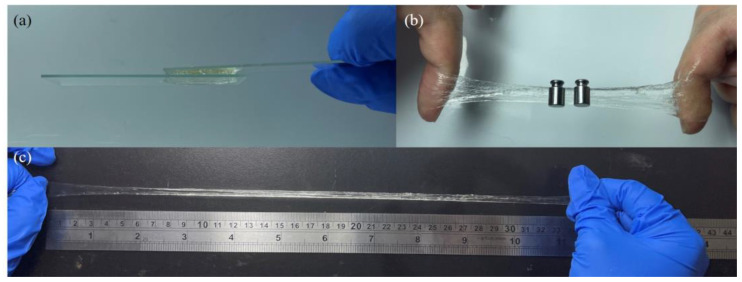
Adhesion, stretchability, and support properties of the PAID-3 hydrogel. (**a**) PAID-3 functions as a glass adhesive, (**b**) PAID-3 adheres to fingers and can successfully support the weight of a weight mass, and (**c**) Stretch testing of PAID-3.

Taking into account both mechanical properties and biocompatibility, we decided to conduct additional adhesion and stretchability tests specifically on the PAID-3 hydrogel [66,67,68]. Our findings indicate that PAID-3 has good adhesion to glass, plastic, stainless steel, gloves, aluminum, and wood, as demonstrated in Figure 8. Due to the adhesive nature of PAID-3 to various materials, we propose a potential interface adhesion mechanism between PAID-3 and the substrate (Figure 9) [69]. Moreover, we measured the adhesive strength of PAID-3 on various substrates and compared it with the literature (Figure S2 and Table S3). Our results demonstrated that PAID-3 exhibited strong adhesive properties, with an adhesive strength of approximately 15.2 kPa on glass. Furthermore, we employed PAID-3 as an adhesive for glass, as illustrated in Figure 10a. By sandwiching PAID-3 between two glass pieces, we observed its effective adhesion to glass surfaces. Additionally, we applied PAID-3 to the fingers of both hands and conducted tests involving tensile forces and weight support (Figure 10b). Remarkably, PAID-3 exhibited exceptional adhesion to the skin, impressive stretchability, and remarkable weight-bearing capabilities. The excellent adhesiveness of PAID-3 to various materials may be attributed to the hydrogen-bonding interactions between AMM and the substrate, as well as the hydrogen bonding and metal coordination between DA and the substrate [70,71]. Figure 10c presents the stretch test result of PAID-3, where the initial size of the hydrogel was 1.5 cm and it demonstrated the ability to stretch over 22 times its original length. Finally, we proceeded to verify the elasticity and toughness of PAID-3. For this purpose, we conducted an experiment where a 500 mL water bottle (filled with water) was supported using PAID-3, and the hydrogel/water bottle was subjected to shaking (Video S1). This test confirmed the excellent resilience and toughness of PAID-3. Consequently, we have successfully developed a hydrogel that combines stretchability, adhesion, and biocompatibility through the synergistic effects of utilizing iron–DA complexes, π-π interaction, and the hydrogen-bond force of DA. This achievement not only holds significant potential for biomedical engineering applications but also for corrosion protection, anti-fouling, and more [72,73,74].

## 4. Conclusions

In summary, we synthesized four hydrogels incorporating AAM, MBA, and DA and investigated the impact of different ratios of iron (III) ions to DA on each hydrogel’s performance. Our research encompassed FT-IR, SEM, tensile stress-strain, and rheology experiments to elucidate the correlation between material properties and intermolecular forces. Through our analysis of PAID-0 and PAID-1, we validated the significance of metal coordination in enhancing the stiffness and toughness of hydrogels. Moreover, by increasing the dopamine content, we achieved improved stretchability and adhesion capabilities in the hydrogels (PAID-1, PAID-2, and PAID-3). Ultimately, we selected the PAID-3 hydrogel with optimal mechanical properties for adhesion testing on various materials. Remarkably, PAID-3 exhibited good adhesion to diverse materials as well as the low cytotoxicity of PAID hydrogel. Overall, it showcased great promise as a new option for biomedical engineering applications.

## Data Availability

All needed data are in the Article.

## Supplementary Materials

The following supporting information can be downloaded at: https://www.mdpi.com/article/10.3390/polym15224378/s1. Table S1: Physical properties of PAID hydrogels; Table S2: Comparison of tensile stress-strain performance between this work and previously reported hydrogels; Table S3: Comparison of adhesive strength between this work and previously reported hydrogels; Table S4: Summarized *t*-test table of *p*-values for cell viability; Figure S1: Degradation curves of PAID hydrogels; Figure S2: Lap-shear strength tests of the PAID-3 hydrogel on different substrates; Figure S3: ^1^H NMR spectrum of methacrylic-modified dopamine in D_2_O; Video S1: Testing of the elasticity and toughness of the PAID-3 hydrogel. References [43,48,75,76,77,78,79,80,81,82,83,84,85,86] are cited in the supplementary materials.
