# Supplementary material for: Stretchable, Adhesive, and Biocompatible Hydrogel Based on Iron–Dopamine Complexes
Source: Polymers (Basel). 2023 Nov 10;15(22):4378. doi: 10.3390/polym15224378 (PMC10674470; doi:10.3390/polym15224378)
Supplement: Supplementary file 1 [file polymers-15-04378-s001.zip › polymers-2614825-supplementary.pdf]

polymers
MDPI

*Supplementary Materials*

# Stretchable, Adhesive, and Biocompatible Hydrogel Based on Iron–Dopamine Complexes

**Celine Lee [1], You-Sheng Zhang [1], He-Shin Huang [1], Yun-Ying Wang [1], Rajan Deepan Chakravarthy [2], Mei-Yu Yeh [1,*], Hsin-Chieh Lin [2,*] and Jeng Wei [3,*]**

[1] Department of Chemistry, Chung Yuan Christian University, No. 200, Zhongbei Rd., Zhongli Dist., Taoyuan City 320314, Republic of China, Taiwan; clys0813@gmail.com (C.L.); candy-huang16@gmail.com (H.-S.H.); g11063013@cycu.org.tw (Y.-Y.W.); brian20010418@gmail.com (Y.-S.Z.)
[2] Department of Materials Science and Engineering, National Yang Ming Chiao Tung University, No. 1001, Daxue Rd. East Dist., Hsinchu City 300093, Republic of China, Taiwan; rdciitmadras@gmail.com
[3] Heart Center, Cheng Hsin General Hospital, No. 45, Cheng Hsin St., Beitou Dist., Taipei City 112401, Republic of China, Taiwan
* Correspondence: myyeh@cycu.edu.tw (M.-Y.Y.); hclin45@nycu.edu.tw (H.-C.L.); jengwei@mac.com (J.W.)

Synthesis of methacrylic-modified dopamine (DA)

Methacrylic-modified dopamine was synthesized following the protocol outlined in the literature [S1]. The synthesis involved the reaction of 3,4-dihydroxyphenethylamine hydrochloride with methacrylate anhydride in an aqueous solution of sodium borate and sodium bicarbonate under moderately basic conditions. The presence of aqueous sodium borate solution, serving as the reaction medium, played a role in protecting the dopamine moiety by forming a borate ester. The synthesized DA monomer was gained as a solid pale-gray powder with a yield of 14%. $^{1}H$ NMR (400 MHz, $D_2O$): δ 1.71 (s, 3H), 2.59 (t, *J*= 6.8 Hz, 2H), 3.31 (t, *J*= 6.8 Hz, 2H), 5.24 (s, 1H), 5.41 (s, 1H), 6.57 (dd, *J*= 2.0, 8.0 Hz, 1H), 6.65 (d, *J*= 2.0 Hz, 1H), 6.72 (d, *J*= 8.0 Hz, 1H).

Table S1 Physical properties of PAID hydrogels.[a]

| | AAM[b] | MBA[b] | $FeCl_3 \cdot 6H_2O$[b] | DA[b] | Appr.[c] | G′, G″ (Pa) |
|---|---|---|---|---|---|---|
| **PAID-0** | 10 | 0.06 | - | 0.18 | TG | $2.51\times10^2$, $1.05\times10^2$ |
| **PAID-1** | 10 | 0.06 | 0.06 | 0.18 | YTG | $1.39\times10^3$, $2.75\times10^2$ |
| **PAID-2** | 10 | 0.06 | 0.06 | 0.36 | YTG | $6.99\times10^2$, $1.53\times10^2$ |
| **PAID-3** | 10 | 0.06 | 0.06 | 0.54 | YTG | $3.52\times10^2$, $0.83\times10^2$ |

[a]Irgacure 2959 (2 mol % to AAM monomer), acrylamide is AAM, *N,N′*-methylene- bis-acrylamide is MBA, and methacrylic-modified dopamine is DA. [b]unit: % w/v; [c]TG: transparent gel; YTG: yellow transparent gel.

Table S2 Compares the tensile stress-strain performance between this work and previously reported hydrogels.

| Materials | Strain (%) | Stress (kPa) | Ref. |
|---|---|---|---|
| **AAM/MAH/$Zn^{2+}$** | 400 | 6000 | S2 |
| **AAM/MBAA/$Cu^{2+}$** | 360 | 550 | S3 |
| **Chitosan/PACG/$Fe^{3+}$** | 550 | 900 | S4 |
| **PMA/$Ca^{2+}$** | 95 | 700 | S5 |
| **MAA/QHPMA/$Zn^{2+}$** | 160 | 580 | S6 |
| **PVA/EGaIn** | 350 | 115 | S7 |
| **AAM/MBA/DA/$Fe^{3+}$** | 1011 | 8 | This work **(PAID-3)** |

Table S3 Compares the adhesive strength between this work and previously reported hydrogels. (substrate: glass)

| Materials | Adhesive strength (kPa) | Ref |
|---|---|---|
| **Aa(Ta)/HMA/AAM** | 0.09 | S8 |
| **PAAM/ PAA/ GR/ PEDOT:PSS** | 2.9 | S9 |
| **AAM/SA/DA** | 5.9 | S10 |
| **PVA/FSWCNT/PDA** | 9.2 | S11 |
| **PVA/PAA/PEDOT:PSS** | 10.97 | S12 |
| **AA/ $ZnCl_2$/AR** | 10.4 | S13 |
| **HACC/PAAM** | 14.4 | S14 |
| **AAM/MBA/DA/$Fe^{3+}$** | 15.2 | This work **(PAID-3)** |

Table S4 Summarized t-test table of p-values for cell viability.

| T-test | p-value (DAY 1) | p-value (DAY 3) | p-value (DAY 7) |
|---|---|---|---|
| **PAID-0 vs Control** | 0.030 | 0.150 | 0.286 |
| **PAID-1 vs Control** | 0.129 | 0.103 | 0.048 |
| **PAID-2 vs Control** | 0.232 | 0.436 | 0.073 |
| **PAID-3 vs Control** | 0.194 | 0.098 | 0.064 |

P-values of $<0.05$ mean that there is a significant difference between the groups.

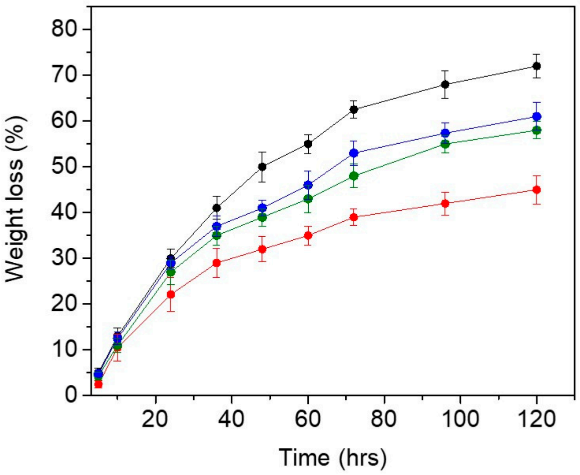


Figure S1: Degradation curves of PAID hydrogels. (Black for **PAID-0**, red for **PAID-1**, olive for **PAID-2** and blue for **PAID-3**)

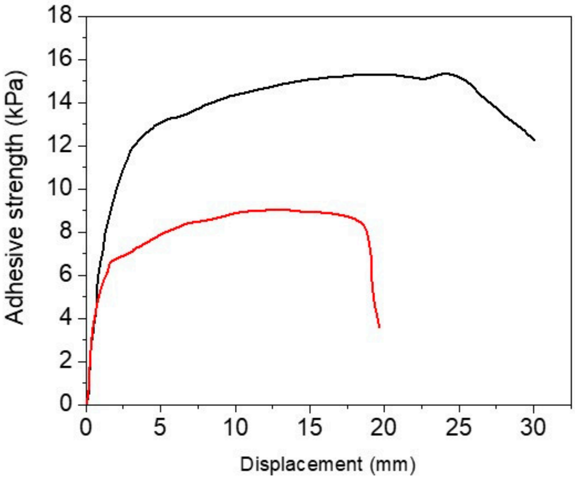


Figure S2: Lap-shear strength tests of the **PAID-3** hydrogel on different substrates. (Black: glass; Red: Al)

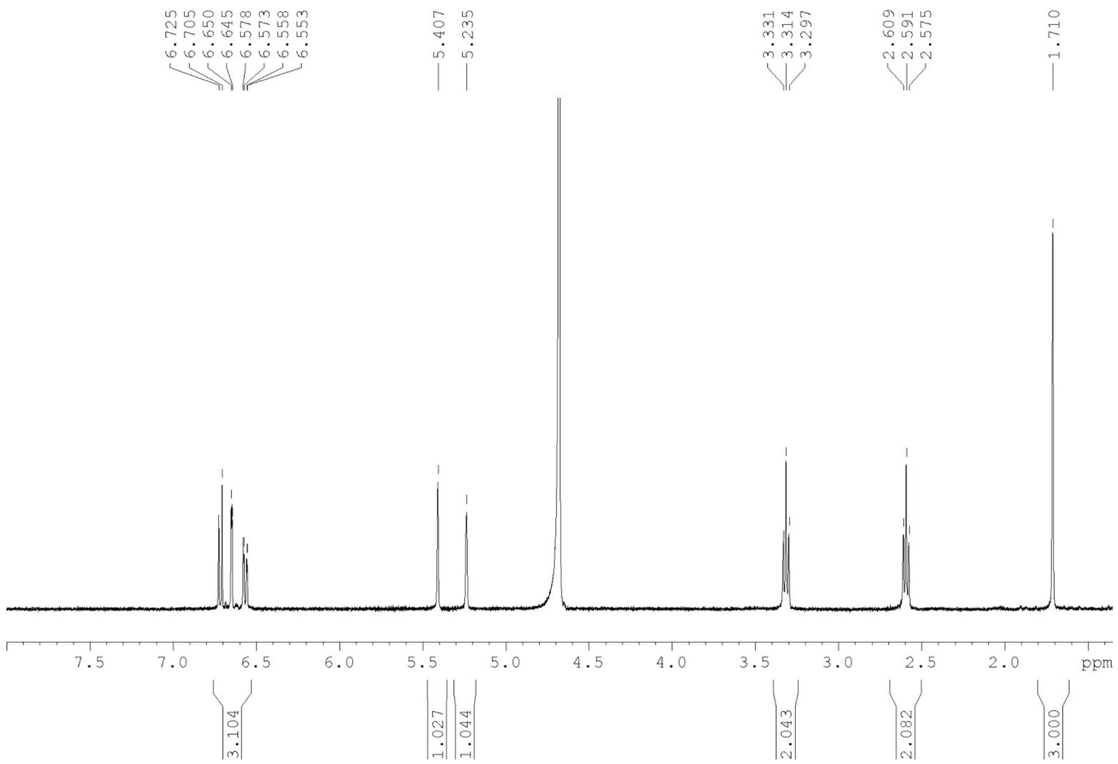


Figure S3: $^1$H NMR spectrum of methacrylic-modified dopamine in $D_2O$.

Reference

[S1] Glass, P.; Chung, H.; Washburn, N. R.; Sitti, M. Enhanced Reversible Adhesion of Dopamine Methacrylamide-Coated Elastomer Microfibrillar Structures under Wet Conditions. *Langmuir* ***2009***, *25*, 6607 -6612.
[S2] Ju, H.; Zhu, Q. L.; Zuo, M.; Liang, S.; Du, M.; Zheng, Q.; Wu, Z. L. Toughening Hydrogels by Forming Robust Hydrazide-Transition Metal Coordination Complexes. *Chem. Eur. J.* **2023**, *29*, e202300969.
[S3] Guo, G.; Sun, J.; Wu, Y.; Wang, J.; Zou, L. Y.; Huang, J. J.; Ren, K. F.; Liu, C. M.; Wu, Z. L.; Zheng, Q.; et al. Tough complex hydrogels transformed from highly swollen polyelectrolyte hydrogels based on Cu(2+) coordination with anti-bacterial properties. *J. Mater. Chem. B* **2022**, *10*, 6414-6424.
[S4] Dou, X.; Wang, H.; Yang, F.; Shen, H.; Wang, X.; Wu, D. One-Step Soaking Strategy toward Anti-Swelling Hydrogels with a Stiff "Armor". *Adv. Sci.* **2023**, *10*, e2206242.
[S5] Zhang, Y.; Wang, R.; Lu, W.; Li, W.; Chen, S.; Chen, T. Mechanical tough and multicolor aggregation-induced emissive polymeric hydrogels for fluorescent patterning. *Nanoscale Adv.* **2023**, *5*, 725-732.
[S6] Zhang, H.; He, J.; Qu, J. High-strength, tough, and anti-swelling Schiff base hydrogels with fluorescent encryption writing, solvent response and double shape memory functions. *Eur. Polym. J.* **2022**, *178*, 111487.
[S7] Wei, Z.; Wang, Y.; Cai, C.; Zhang, Y.; Guo, S.; Fu, Y.; Tan, S. C. Dual-Network Liquid Metal Hydrogel with Integrated Solar-Driven Evaporation, Multi-Sensory Applications, and Electricity Generation via Enhanced Light Absorption and Bénard–Marangoni Effect. *Adv. Funct. Mater.* **2022**, *32*, 2206287.
[S8] Zhang, Q.; Liu, X.; Duan, L.; Gao, G. Ultra-stretchable wearable strain sensors based on skin-inspired adhesive, tough and conductive hydrogels. *Chem. Eng. J.* **2019**, *365*, 10-19.
[S9] Dong, L.; Wang, M.; Wu, J.; Zhu, C.; Shi, J.; Morikawa, H. Stretchable, Adhesive, Self-Healable, and Conductive Hydrogel-Based Deformable Triboelectric Nanogenerator for Energy Harvesting and Human Motion Sensing. *ACS Appl. Mater. Interfaces* **2022**, *14*, 9126-9137.
[S10] Chen, T.; Chen, Y.; Rehman, H. U.; Chen, Z.; Yang, Z.; Wang, M.; Li, H.; Liu, H. Ultratough, Self-Healing, and Tissue-Adhesive Hydrogel for Wound Dressing. *ACS Appl. Mater. Interfaces* **2018**, *10*, 33523-33531.
[S11] Liao, M.; Wan, P.; Wen, J.; Gong, M.; Wu, X.; Wang, Y.; Shi, R.; Zhang, L. Wearable, Healable, and Adhesive Epidermal Sensors Assembled from Mussel-Inspired Conductive Hybrid Hydrogel Framework. *Adv. Funct. Mater.* **2017**, *27*, 1703852.
[S12] Peng, X.; Wang, W.; Yang, W.; Chen, J.; Peng, Q.; Wang, T.; Yang, D.; Wang, J.; Zhang, H.; Zeng, H. Stretchable, compressible, and conductive hydrogel for sensitive wearable soft sensors. *J. Colloid Interface Sci.* **2022**, *618*, 111-120.
[S13] Fu, Q.; Hao, S.; Meng, L.; Xu, F.; Yang, J. Engineering Self-Adhesive Polyzwitterionic Hydrogel Electrolytes for Flexible Zinc-Ion Hybrid Capacitors with Superior Low-Temperature Adaptability. *ACS Nano* **2021**, *15*, 18469-18482.
[S14] Wang, L.; Zhou, M.; Xu, T.; Zhang, X. Multifunctional hydrogel as wound dressing for intelligent wound monitoring. *Chem. Eng. J.* **2022**, *433*, 134625.

**Disclaimer/Publisher's Note:** The statements, opinions and data contained in all publications are solely those of the individual author(s) and contributor(s) and not of MDPI and/or the editor(s). MDPI and/or the editor(s) disclaim responsibility for any injury to people or property resulting from any ideas, methods, instructions or products referred to in the content.
